# A microgripper based on electrothermal Al–SiO_2_ bimorphs

**DOI:** 10.1038/s41378-024-00821-2

**Published:** 2024-12-16

**Authors:** Hengzhang Yang, Yao Lu, Yingtao Ding, Ziyue Zhang, Anrun Ren, Haopu Wang, Xiaoyi Wang, Jiafang Li, Shuailong Zhang, Huikai Xie

**Affiliations:** 1 School of Integrated Circuits and Electronics, Beijing Institute of Technology, Beijing, China; 2 Engineering Research Center of Integrated Acousto-opto-electronic Microsystems, Ministry of Education of China, Beijing, China; 3 Chongqing Institute of Microelectronics and Microsystems, Beijing Institute of Technology, Chongqing, China; 4 School of Physics, Beijing Institute of Technology, Beijing, China; 5 Zhengzhou Research Institute, Beijing Institute of Technology, Zhengzhou, China

**Keywords:** Electrical and electronic engineering, NEMS

## Abstract

Microgrippers are essential for assembly and manipulation at the micro- and nano-scales, facilitating important applications in microelectronics, MEMS, and biomedical engineering. To guarantee the safe handling of delicate materials and micro-objects, a microgripper needs to be designed to operate with exceptional precision, rapid response, user-friendly operation, strong reliability, and low power consumption. In this study, we develop an electrothermal actuated microgripper with Al-SiO_2_ bimorphs as the primary structural element. The fabricated microgripper naturally adopts a closed state due to process-induced residual stresses. The thermal expansion mismatch between Al and SiO_2_ allows for an easy transition of the microgripper between open and closed states by temperature control. Experimental data reveal that the microgripper can achieve impressive deformability, bending over 100 degrees at just 5 V, and responding within 10 ms. Its capability to handle micro-objects is verified using polymethyl methacrylate (PMMA) microbeads and its gripping strength is quantitatively assessed. It is demonstrated that the microgripper holding a microbead with a diameter of 400 μm and a weight of 0.1 mg can withstand an average acceleration of 35 *g* during vibration test and over 1600 *g* in impact tests, highlighting its exceptional grasping performance. Additionally, the “pick-and-place” task for handling and positioning solder beads (0.25 mg for each bead) with diameters of 400 μm on a bulk silicon inductor chip has been successfully completed. This unique microgripper is anticipated to be highly beneficial for various micro-assembly and micromanipulation applications, particularly in the field of electronic packaging.

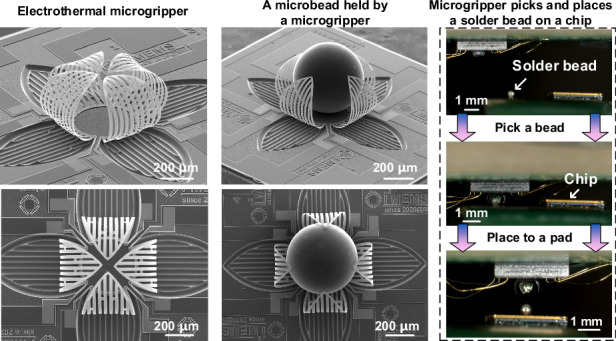

## Introduction

Microgrippers are capable of manipulating and handling extremely small objects, typically on the micrometer scale. Due to their compact size and significant deformation capability, microgrippers have attracted widespread attention and interest. They have been extensively researched for various applications, including biopsy procedures, where they are used to remove diseased tissue from patients for pathological examination, aiding clinicians in diagnosing diseases^1–3^; in 3D programmable assembly, where they enable the assembly of structures in constrained or complex environments^4–6^; and in the manipulation of microparticles, such as in targeted drug delivery tasks^7,8^ and transportation in challenging environments^9–11^. Typically, a microgripper consists of two or more actuators that deform in response to external stimuli. Numerous methods have been developed for manufacturing micro-actuators, using various mechanisms, such as electrostatic^12–16^, electrothermal^17–19^, electromagnetic,^20–22^ optical^23,24^ and chemical^25,26^ actuation. For instance, O. M. Wani *et al*. developed an optical-driven microgripper capable of capturing millimeter-scale micro-cubes weighing 10 mg through precise control of light path and power^27^. J. Shintake et al. designed a millimeter-scale soft electrostatic microgripper, which achieved a bending angle of 30° under a 5 kV driving voltage with a response time of 100 ms^28^. Additionally, J. Zhang et al. introduced a force-controlled magnetic microgripper designed for micro-scale manipulation and characterization, achieving a rapid response time of 25 ms^29^. These microgrippers are important in advancing precision engineering and microscale technologies, enabling effective manipulation and assembly of small-scale components on demand.

Despite the good performance demonstrated by the aforementioned microgrippers, several technological challenges and limitations still exist. Light-driven microgrippers typically require specific light sources with certain power and wavelength requirements to ensure effective light absorption. Additionally, they necessitate carefully designed optical paths to facilitate effective interactions between the light beam and the actuator. Electrostatic-driven microgrippers generally require the application of high voltage (ranging from hundreds to even thousands of volts) to the actuator, which may pose safety issues and increase the complexity of system operation. Electromagnetic-driven microgrippers involve the use of magnetic-responsive materials and complex systems to generate programmable magnetic fields, and are also challenging to deploy in narrow and enclosed microenvironments. Therefore, developing high-performance, small-form-factor, user-friendly, and widely applicable microgrippers is still of great significance and in great need.

**Fig. 1:**
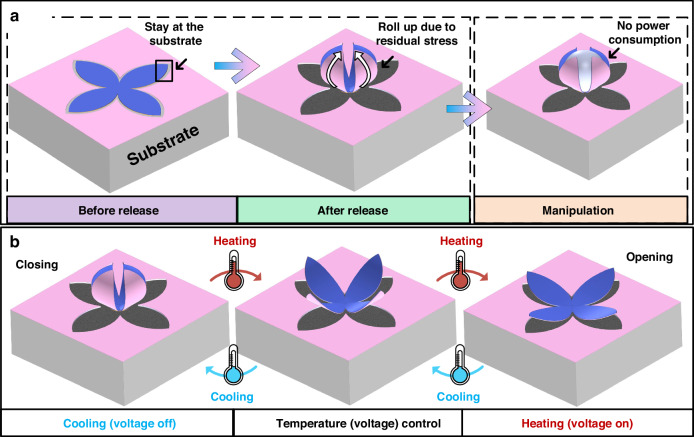
The concept of the microgripper. **a** The formation of curved actuators based on the utilization of residual stresses. **b** The operating method of the microgripper

Compared to many other actuation mechanisms, electrothermally driven microgrippers offer the advantage of achieving significant structural deformation with a simple structure and low driving voltage^29–31^. In this study, we developed an electrothermal driven microgripper characterized by large reversible deformation, fast response, ease of operation, robust grasping strength, and high stability. These features are crucial for broadening the range of applications for microgrippers. Figure 1 illustrates the structure and working mechanisms of the electrothermal-driven microgripper, which is inspired by the closing and opening of flower petals. This microgripper utilizes electrothermal bimorph actuators as the main structure. Each electrothermal bimorph consists of two materials that have a large coefficient of thermal expansion (CTE) difference. These petal-shaped actuators all bend down in the closed state of the microgripper, which can realize a full holding of a small object after capturing it. The bimorphs can be released by undercutting the silicon beneath and residual stresses in the bimorph result in an initially closed state of the microgripper, as shown in Fig. 1a. In addition, thanks to the residual stresses, the microgripper won’t consume any power when capturing samples in the closed state (as Fig. 1a shows). During manipulation tasks, it is easy to switch between two operating modes—opening and closing—by switching on and off the voltage applied to the bimorph actuators. Additionally, the extent of deformation can be precisely controlled by adjusting the amplitude of the driving voltage, as illustrated in Fig. 1b. To achieve individual control of all the petals of a microgripper, a separate resistive heater is embedded in each petal. A preliminary study on this device was presented by Yang et al.^32^ This work further investigates various aspects of the microgripper, including the device design, actuator control, micro-object manipulation and reliability tests. PMMA beads of different diameters are used to characterize the microgripper’s grasping ability and grasping strength. In terms of application, the microgrippers proposed in this paper are employed to transport and position the solder beads on inductor chips, showing their great potential in the field of electronic packaging.

## Methodology

### Design of the microgripper

**Fig. 2:**
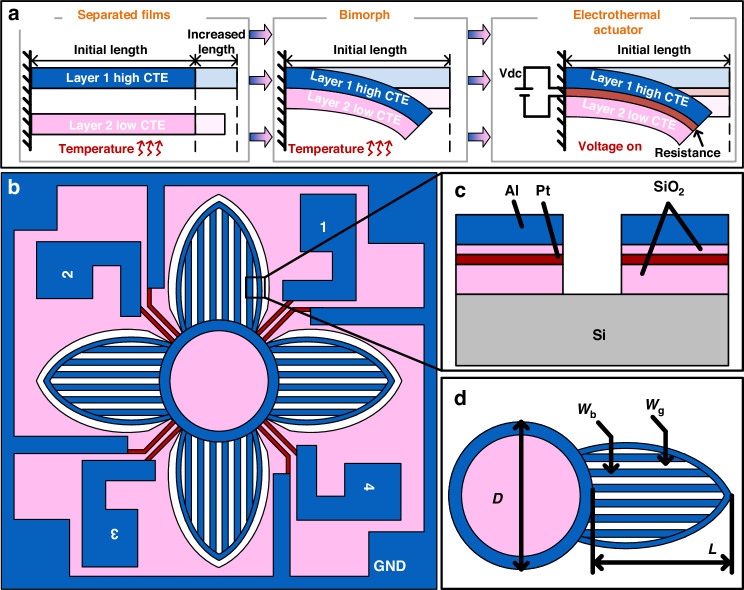
Design of the electrothermal microgripper. **a** Working principle of the electrothermal bimorph actuator. **b** Topology design. **c** Cross-sectional view of two adjacent bimorphs. **d** Enlarged view of an actuator showing the key structural parameters. *D*: diameter of the center plate. *L*: length of the actuator. *W*_b_: width of the bimorph. *W*_g_: width of the release gap

Figure 2a illustrates the working mechanism of the electrothermal driving method, wherein two materials with distinct CTEs are bounded together to create a bimorph actuator. When a temperature load is applied to the bimorph, the two materials undergo different thermal deformation, thus producing a stress mismatch and subsequently leading to the structural bending of the actuator. Embedding a resistor within the bimorph actuator for heating enables the conversion of electrical energy into thermal energy, resulting in the mechanical structural change of the micro-actuator.

The release rate of the device is an important parameter. Typically, isotropic etching via XeF_2_ or SF_6_ is used to remove the silicon beneath the bimorphs. A perforated bimorph structure is employed to increase the release rate of the bimorph actuators. Specifically, each bimorph actuator is made of an array of bimorph beams with different lengths. Figure 2b shows the optimized layout design of the microgripper. Four symmetrical actuators are distributed around the center plate, which are designed to have scalloped patterns to ensure an excellent closing performance of the microgripper. Each actuator is independently controllable via applying voltage to a specific contact pad, allowing the microgripper to have multiple driving modes. In addition, perforated structures are used to increase the release rate of the devices, and a simulation is conducted to confirm the relationship between the curling direction and bimorph angles (as shown in Fig. S2). Note that all of the holes are identical to ensure a uniform etch during the release. Figure 2c shows a cross-sectional view of the actuator. To maximize the deformability, Al and SiO_2_ are used to fabricate the bimorph actuators due to their large CTE difference (as shown in Table S1). A serpentine-shaped Pt resistor is embedded between Al and SiO_2_, and a thin layer of SiO_2_ insulation layer is used to ensure the electrical isolation between Al and Pt so that the Pt resistor can generate Joule heat effectively. Pt and Al conductive wires are electrically interconnected through the patterned SiO_2_ surface on the substrate. Figure 2d shows the key structural parameters of the actuator, and the detailed values are listed in Table S2.

### Fabrication process flow

**Fig. 3:**
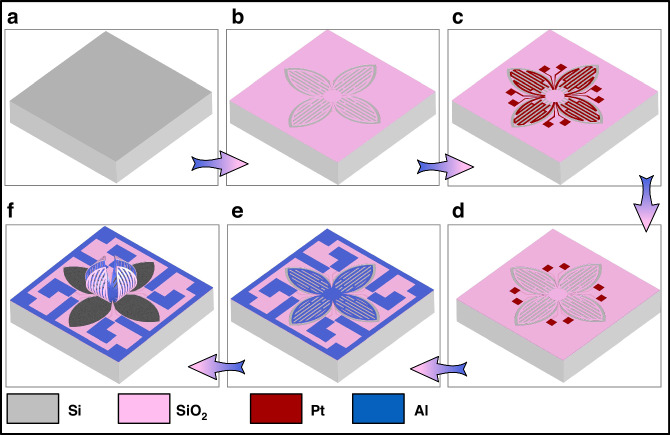
Fabrication process steps. **a**, **b** PECVD-based SiO_2_ depostion and dry etch. **c** Sputtering Pt & lift-off. **d** PECVD SiO_2_ deposition and dry etch. **e** Sputtering Al and dry etch. **f** Device release

A unique microfabrication process is implemented on a Si wafer to fabricate the microgripper, as demonstrated in Fig. 3. Four photolithography steps are needed during the whole fabrication process. Initially, a 400 nm-thick SiO_2_ layer is deposited on the Si wafer via Plasma-Enhanced Chemical Vapor Deposition (PECVD) at 300 °C, followed by a Reactive Ion Etching (RIE) process for patterning. Subsequently, a 100-nm-thick Pt layer is sputtered and then patterned by lift-off. Following this, a 100-nm-thick SiO_2_ layer is deposited to ensure electrical insulation between Pt and Al, concurrently defining the connection holes distributed on the substrate. After that, a 500 nm thick Al layer is deposited and patterned via RIE, delineating both the actuators and the electrical wires on the substrate. Finally, SF_6_ gas is employed to etch the Si beneath the actuators, and the release step is rapidly accomplished within 5 min due to the specifically designed holes.

## Results

### Fabricated devices

**Fig. 4:**
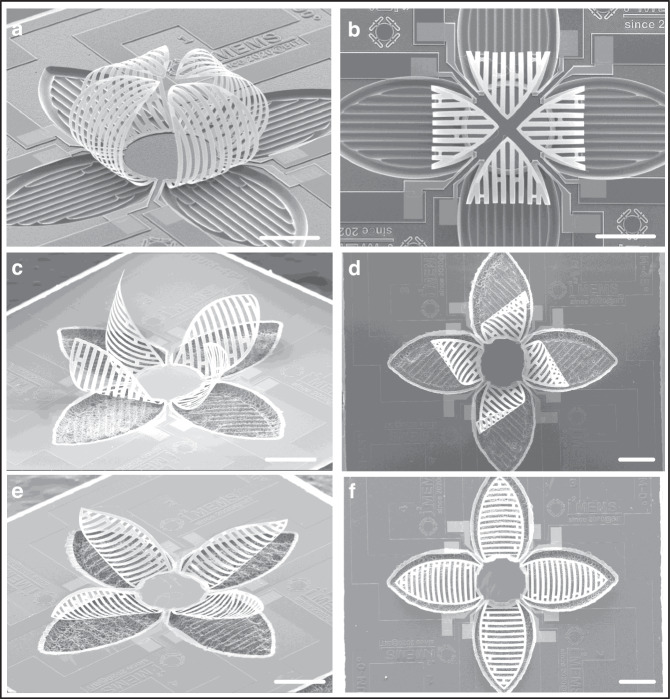
SEM images of fabricated microgrippers. **a**, b Bimorph beams are arranged along the longitude direction. **c**, **d** Bimorph beams are arranged along a 45-degree direction. **e**, **f** Bimorph beams are arranged along the latitude direction. The scale bar in all figures are 250 μm

The SEM images in Fig. 4a, b are taken from a fabricated microgripper, showing the uniform and smooth curling of all four actuators due to residual stresses. In addition, four actuators form a spherical space after release, which is suitable for capturing and holding micro-objects. Devices with bimorph beams arranged at a 45-degree angle direction (as shown in Fig. 4c, d) and along the latitude direction (as shown in Fig. 4e, f) have also been fabricated. Notably, despite the three actuators having the same contour shape, different orientations of the bimorph beams result in significantly different deformation states. A reasonable explanation is that the orientation of the bimorph beams determines the direction of the residual stress acting on the actuator and control the actuators’ stiffness along the direction of the bimorph beams, thereby influencing the structure state after release. Fig. S1 shows the images of microgrippers arrays, which have good structural consistency for each unit. It is expected that the microgripper array will be useful for tasks requiring parallel control and manipulation of multiple targets simultaneously.

### Actuation characteristics of the microgripper

**Fig. 5:**
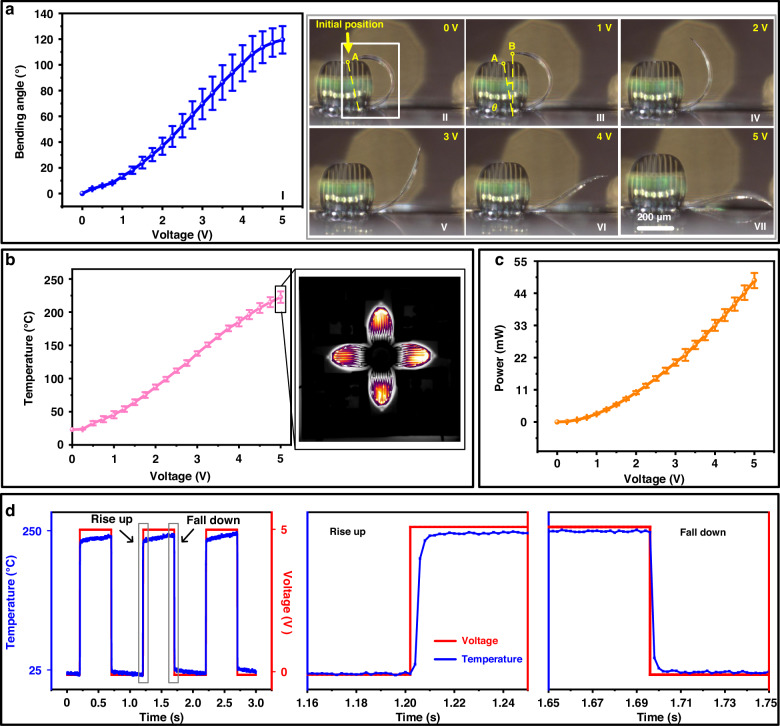
Actuation characteristics of the microgrippers. **a**(I) Profile of the bending angle versus applied DC voltage for the actuators. **a**(II–VII) Shapes of an actuator at different driving voltages. **b** Profile of the average temperature versus applied DC voltage for the actuators. **c** Profile of the DC power versus applied DC voltage for the actuators. **d** Curves of the thermal response time of the microgripper

After device fabrication, the microgripper is fixed on a Printed Circuit Board (PCB) board to test its characteristics, where the electrical connection between the device and the PCB board is achieved by wire bonding. In this study, the performances of ten actuators were characterized. The resistances of the ten testing actuators are shown in Table S3. Initially, the actuator is in the closing state without power input. When the driving voltage is set from 1 V to 5 V, the bending angle of the actuator gets larger with the increase of the driving voltage in an approximately linear trend (between 1.5 V to 4.5 V). The bending angle is defined as θ in Fig. 5a(III). When the voltage reaches 5 V, the actuator reaches a fully expanded state and the opening angle of the tip exceeds 100 degrees. Figure 5a(II–VII) shows the deformation process of a representative actuator with the driving voltage changing from 0 V to 5 V. Note that the actuators can precisely bend to and maintain a desired angle by finely modulating the driving voltage. As mentioned before, the four actuators can be controlled individually and independently to meet the specific capturing requirements. Figure S3a–e illustrates the results where the four actuators of the microgripper are driven in sequence at 5 V. Moreover, the simultaneous activation of all the four actuators is also feasible. Figure S3f–j depicts the deformation procedure of a microgripper while the driving voltage on all the actuators ranges from 1 V to 5 V. It is estimated that the breakdown voltage of the actuator is around 8 V.

The mean operational temperatures of the actuators are closely monitored utilizing the temperature coefficient of resistance (TCR). The detailed measurement procedure of TCR is elucidated in Fig. S4. The TCR value of Pt is firstly quantified via a temperature-regulated chamber to be approximately 0.0026/°C. After that, by monitoring the alterations in the resistance of Pt at different driving voltages, the mean temperature of an actuator can be obtained. The correlation between the average working temperature and applied voltage is depicted in Fig. 5b. At 5 V, the average working temperature of the actuators stays at approximately 220 °C. The temperature distributions of the actuators are also characterized using an infrared thermal imaging system (ImageIR 8355B), and a representative result is shown in the inset of Fig. 5b. Notably, due to the absence of a thermal isolation structure between the actuator and the substrate, heat concentration is observed primarily at the actuator tips, while the root-part remains inadequately heated, thus exhibiting significant temperature gradients on the actuator. Furthermore, the power consumptions of the actuators are measured and presented in Fig. 5c. The average power consumption per actuator is approximately 48 mW when operating at the fully opening state.

To achieve fast capturing, the response speed of the actuator is a key parameter. In this work, both the thermal response time and mechanical response time are measured and quantified. Temperature changes of the actuators over time are monitored by an infrared thermal camera. In the tests, a continued square wave signal is applied to the actuators. The peak-to-peak voltage value is 5 V, and the frequency is set at 1 Hz. The temperature evolution over time is shown in Fig. 5d. The enlarged views of the rise and fall periods are also provided. Consequently, The structure change of a petal actuator depends on its electro-thermo-mechanical response. The measured response times for the opening and closing processes, i.e., the rise time and fall time, are shown in Fig. 5d, which are approximately 8 ms and 10 ms, respectively. Besides, the mechanical response time is assessed using a high-speed camera, capturing images at a rate of 2000 frames per second (f/s). The opening and closing processes of the microgripper are shown in Fig. S5 and Fig. S6, respectively. Additionally, the detailed deformation process of the microgripper is shown in Supplementary Movie S1, where the playback speed is 1/30 of the actual speed.

### Capturing experiment

**Fig. 6:**
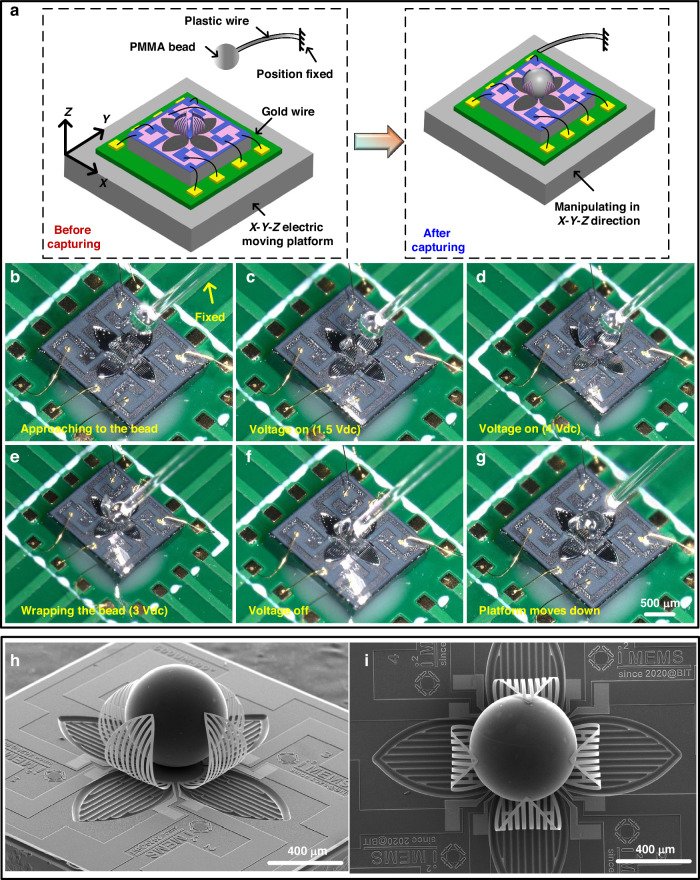
Manipulation process. **a** Schematic illustration of the experiment setup. **b**–**g** Step-by-step manipulation process. **h**, **i** SEM images of the microgripper after capturing the microbead

A specialized capturing experiment is conducted to assess the functionality of the microgripper. A schematic illustration of the setup and the test is shown in Fig. 6a. Initially, the microgripper is attached to a PCB board using Ultra-Violet Ray (UV) glue, with four actuators electrically connected to the PCB board via gold wires. Subsequently, a PMMA microbead with a diameter of 500 μm is adhered to a plastic wire by electrostatic force. The mass of each microbead is measured to be 0.12 mg and the plastic wire is fixed to make the bead static. Then the microgripper is positioned on an *XYZ* stage with a movement accuracy at the micron level.

Figure 6b–g shows the detailed manipulation process of the microgripper. First, the platform moved upward to bring the device close to the microbead (Fig. 6b). Then, DC voltages are applied to the four actuators and gradually increased to ensure the opening is large enough to wrap the microbead, and then precisely control the position of the platform within the plane so that the microgripper can accurately touch and capture the microbead (Fig. 6c, d). Next, the platform is further lifted precisely to achieve an accurate contact between the device and the microbead (Fig. 6e). The applied voltages are then gradually diminished until zero, closing the actuators accordingly under the influence of residual stresses (Fig. 6f). Finally, the platform moves downward. As depicted in Fig. 6g, the device successfully captures the microbead and takes it from the plastic holder. Figure 6h, i shows the SEM images of the microgripper after capturing. Despite that only the tips of the actuators contact with the surface, the captured microbead is effectively grasped by the microgripper. In addition, we conducted more experiments aiming to capture multiple targets using a single microgripper, and the results are presented in Fig. S7.

### Reliability test

**Fig. 7:**
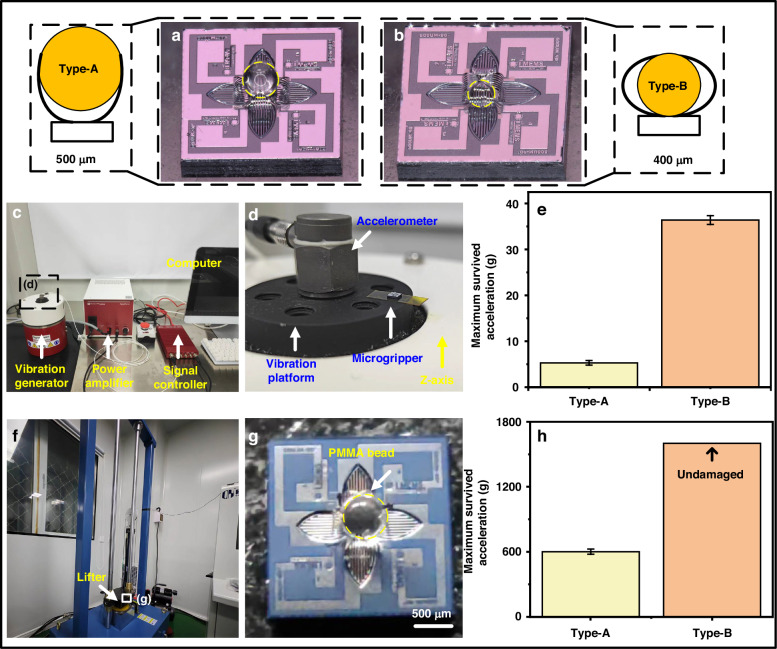
Reliability tests. **a**, **b** Two wrapping modes of the microgripper. **c**, **d** Detailed setup of the vibration test. **e** Vibration test results of the two wrapping modes. **f**, **g** Detailed setup of the impact test. **h** Impact test results of the two wrapping modes

In the actual micro-manipulation process, the microgripper may be affected by environmental vibrations or accidental collisions, which may cause physical damage to the actuators, resulting in the falling of the captured object and mission failure. Therefore, the microgripper’s grasping strength needs to withstand environmental disturbances. In this study, a vibration table (SignalCalc 901 DP) and an impact testing system (OK-S-10) are used to study how strongly the microgripper can grasp and hold a small object. Figure 7a, b illustrates the initial capturing scenarios of two kinds of PMMA microbeads with diameters of 500 μm and 400 μm, respectively. In these cases, two different wrapping modes including half wrapping (defined as Type-A) and full wrapping (defined as Type B) are observed. Then, the ability of the devices to resist physical vibration is evaluated by a vibration test system (SignalCalc 901 DP), as shown in Fig. 7c. The vibration of the platform is triggered by a sinusoidal signal with varying frequency and amplitude. Following the JESD22-B103B standard30^33^, the vibration frequency is set to sweep from 20 to 2000 Hz and then back to 20 Hz, which is increased on a logarithmic scale with the duration set to 12 min. For each frequency sweep, the acceleration amplitude is incrementally raised by 1 g until the captured-bead bounces off from the microgripper. Throughout the entire vibration test, the status of the microgripper can be directly observed by the naked eyes. The microgripper is affixed to the platform using a double-sided adhesive tape, where the platform has only one freedom of movement in the *Z* direction as depicted in Fig. 7d. Figure 7e displays the results of the vibration tests. For Type A, the maximum endurable acceleration is approximately 5 *g*, whereas it is about 35 *g* for the Type B. A detailed failure process of Type A is shown in supplementary Movie S2. The condition of the microgripper is monitored both before and after the vibration test, and the results are depicted in Fig. S8. Despite the bead detaching from the microgripper due to vibration, the overall shape and functionality of the device remain unaffected. That is, under the influence of residual stress, the actuators are capable of recovering to the initial closing state. Furthermore, upon the reapplication of the excitation signal to the microgripper, the actuators can resume normal operations.

Figure 7f, g shows the setup of the impact test. Two types of the microgripper are respectively affixed to the surface of the lifting platform using double-sided tape. Precise control over the impact experiment’s height is achieved utilizing a control system. The lifting platform is equipped with an acceleration sensor, enabling real-time monitoring of the impact acceleration during descent and collision via a computer interface. The equipment is capable of generating a maximum acceleration of 1600 g, corresponding to a drop height of 1.3 meters on the lifting platform. In each impact test, the drop height is increased by 5 cm until the microbead falls from the microgripper. The test results are shown in Fig. 7h. For Type A, the maximum endurable acceleration is approximately 600 *g*, while for Type B, the bead remains securely grasped by the microgripper even under an impact acceleration of 1600 *g*. We also evaluated the structural change of the microgripper after the impact tests, and the results are shown in Fig. S9. Fig. S9a–c illustrates the morphology of the device for Type A after the tests, where the microgripper does not revert to its initial closing state. A reasonable assumption would be that upon experiencing an instantaneous impact, the PMMA microbead generates a strong collision with the actuators. Given that the entire microbead is constrained solely by the tips of the actuators, external impact readily breaks the constraint. Additionally, the impact of the PMMA microbead induces a certain degree of irreversible deformation of the actuators. In contrast, when the PMMA microbead is fully wrapped by the microgripper, our study indicates that the influence of the microbead on the microgripper is small and with little effect on the residual stress of the actuators. Consequently, the microgripper remains in a closing state, merely indistinguishable from its initial state upon release. Subsequent to the impact, the captured microbead slightly shifts its position within the microgripper, as illustrated in Fig. S9d–f. These results demonstrate the superior performance of the microgripper to withstand strong vibration and physical impact.

## Manipulation test

**Fig. 8:**
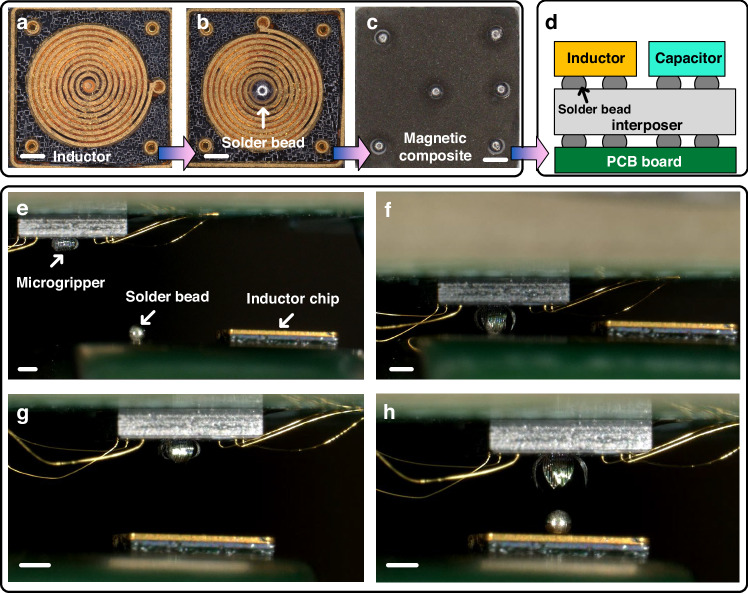
Potential applications use of microgrippers in electronic packaging. **a**–**c** Micrgrippers complete the handling and alignment of solder beads on the surface of inductors. **d** Schematic diagram of microgrippers completing solder bead handling and alignment in a small system. **e**–**h** Manipulation process of the microgripper. All scale bars in Fig. 8 are 500 μm

At present, ball grid array (BGA) technology has become one of the mainstream technologies for integrated circuit packaging. Compared with wire bonding technology, BGA packaging technology greatly reduces the length of interconnection lines and effectively increases packaging density. In the packaging process, the precise alignment of solder beads and pads greatly affects the performance and reliability of the circuit system. The steel mesh bead planting method is an effective way to achieve alignment between solder beads and pads^34^. Although this method can achieve large-scale alignment, its heavy dependence on masks makes it difficult to play its advantages when facing the bead planting of a single device or the bead planting between multiple devices with pads of various sizes. High-precision laser bead planting equipment can achieve efficient maskless bead planting^35^, but the equipment is expensive and requires well-trained personnel. Using mass-produced microgrippers to carry and transfer solder beads is a feasible way to achieve maskless and low-cost bead planting. It can be used for the bead planting of a single device or a small system with various pad sizes. Figure 8a–d shows a feasible case. First, microgrippers are used to complete the handling and positioning of solder beads on a single device. After the single device is processed, the handling and positioning of solder beads on the micro-system can be completed, thereby assisting in the entire packaging work.

In our case, microgrippers based on Al-SiO_2_ Bimorph are used to excellently complete the task of handling and positioning solder beads with a diameter of 400 μm on the bulk silicon inductor chip. The measured mass of the solder beads was 0.25 mg each. Figure 8e–h shows the detailed process of the operation. The detailed workflow can be found in the supplementary materials. First, the microgripper is fixed on a plate that can move in the X-Y-Z direction. Then the translation stage is controlled to apply voltage to the actuators while approaching the solder bead, and the microgripper is controlled to open to a suitable size. When the microgripper contacts the solder bead, the voltage is gradually reduced. Under the influence of residual stress, the opening angle of the microgripper gradually decreases until the solder bead is completely wrapped. Afterwards, the plate is controlled to transfer the microgripper to the top of the chip solder joint, and a DC voltage is applied to the actuator to open the microgripper. The solder bead falls to the chip solder joint, completing the transfer process In this experiment, the resistance of the four actuators of the microgripper is similar. After the four actuators are connected in parallel, the deformation angles of the actuators can be controlled to be similar. It is worth noting that when the resistances of the four actuators are different, since each actuator can be controlled independently, the voltage applied to each actuator can be fine-tuned to control their deformation angles to be similar. The number of gripping times is tested in this study to evaluate the durability of the microgripper. The experimental information is shown in Fig. S10, Video S5 and S6. The experimental results show that for samples with a diameter of less than 400 μm, the microgripper can work around 10 thousand times. For samples with a diameter greater than 400 μm, such as the PMMA microbeads shown in Fig. 6, the microgripper can work around 100 thousand times.

## Discussion

In the previous sections, we introduced the layout design, fabrication process, actuating characteristics, capture capability, and grasping strength of the microgripper, which shows many advantages of the microgripper, such as easy-to-operate, large structure deformation, fast response, and strong capability to withstand vibration and impact. However, some points are worth to be carefully considered for improvements. For example, it is necessary to reserve large space between adjacent actuators to accommodate the routing of interconnect lines and minimize the influence of isotropic etching on the device fabrication (as shown in Fig. 3). This necessity results in significant gaps between neighboring actuators, presenting a challenge in the capture of even smaller micro-scale objects. To solve this problem, the through silicon via (TSV) technology could be effectively cooperated with the microgripper, and the interconnect lines could be routed out through the center plate by TSVs, thus the whole device would be much more integrated. Besides, it is interesting to note that when the applied voltage reaches 5 V, the actuator cannot fully contact the substrate, showing a shape similar to a suspension bridge (as shown in Fig. 5(a)(VII)). This is due to the lack of a thermal isolation structure at the root of the actuator, resulting in the heat dissipating into the substrate and subsequently causing an uneven heat distribution to form a suspension bridge-like structure. For the same reason, even if only a single actuator is driven, Joule heat can still act on other actuators through heat transfer, and this thermal crosstalk phenomenon is clearly illustrated in Fig. S3a–e). More detailed information on the thermal crosstalk is shown in Fig. S11. Thanks to the low thermal conductivity, good thermal isolated materials such as SiO_2_ inserted between the actuator and the substrate may solve this problem. Moreover, the power consumption can also be further reduced by choosing the proper material combination and thicknesses of the bimorph layers, which can be beneficial for multiple bead planting applications as mentioned in Fig. 8. Note that only the opening state causes power consumption, which means the targets can stay in the microgripper for a long time without any power consumption. Therefore, this device can also be utilized in the application of long-term holding and preservation of samples.

Compared with the previously reported electrothermal microgripper summarized in Table S4, instead of beam-like structures, this actuator adopts a petal-like structure, which can achieve complete wrapping of target objects. For the electronic packaging application shown in Fig. 8, this is beneficial for capturing smooth solder beads of various sizes. A microgripper carrying solder beads of different sizes is shown in Video S3 and Video S4. These experiments show that the microgripper can perform micro-manipulation on solder beads with diameters of 200–500 μm. In addition, the four actuators of the microgripper can be controlled independently, which gives the microgripper more flexible deformation capabilities.

Also, the working temperature of the actuators is another key parameter that needs to be carefully considered. In our case, the average temperature can reach over 200 °C at 5 V. On the one hand, repeated switching operations at high temperatures will accelerate the aging of the actuator, thereby reducing its working life. Also, excessively high temperatures may cause irreversible damage to the captured samples, such as organic life samples and metal samples with low melting points. Generally, polymer materials have a high coefficient of thermal expansion. Combining them with materials with a low coefficient of thermal expansion to form a bimorph can effectively reduce the operating temperature of the actuator.

During the human-robot interaction, the Al-SiO_2_ actuators are easy to fail due to physical impacts and collisons. For instance, in the impact test for Type A, an actuator is struck by a PMMA microbead, resulting in irreversible deformation. The microgripper introduced in the work is an actuator made of Al and SiO_2_. Although the large difference in thermal expansion coefficient between the two bimorph materials can bring advantages to actuator deformation, SiO_2_ itself is a brittle material and is easily broken by impact, which limits the reliability of the device. We will consider using other soft materials with small thermal expansion coefficients to replace SiO_2_. Fig. S12 shows that some microgrippers are damaged due to accidental bumps during the experiment. To enhance the actuator reliability and versatility across a range of target sizes and materials, as well as to improve the device’s performance to handle environmental disturbances, the utilization of polymer materials with low Young’s modulus, such as polyimide and hydrogels, should be taken into consideration. These materials exhibit good deformability to alleviate stress upon impact and may potentially improve the reliability of the microgripper. Generally, polymer materials, e.g., photosensitive polyimide, have high CTE, as shown in Table S1. Combining a high-CTE polymer with a low-CTE material to form a bimorph can effectively reduce the operating temperature of the actuator. However, high-CTE polymers usually have low thermal conductivity, which on one hand can increase the heating efficiency but on the other hand will increase the thermal response time of the bimorph. Thus, the material types, layer structures and layer thicknesses must be all considered to optimize the overall performance of bimorph-based devices.

## Conclusion

In this study, an electrothermal driven microgripper has been developed. By employing a standard MEMS fabrication process with commonly used materials, the designed microgripper demonstrates promising prospects for large-scale manufacturing. The advantages of the low driving voltage, large deformation, and fast response make the microgripper a very useful tool for micromanipulation applications. Moreover, the microgripper has shown excellent capturing capability and reliable grasping strength, verified by strong vibration and impact tests. It is expected the microgripper demonstrated in this work will be of great use for a variety of microassembly and microfabrication applications in which precisly capture and release of micro-targets is in great need.

## Supplementary information

Supplemental Material File #1

Actuating Process of the microgripper

Vibration test of the microgripper

Solder bead micro assembly process with a diameter of 400μm

Solder bead micro assembly process with a diameter of 200μm

long-time driving test of the microgripper

long-time driving test of the actuator

## References

[1] Gultepe, E. et al. Biopsy with thermally-responsive untethered microtools. *Adv. Mater.* **25**, 514–519 (2013). 23047708 10.1002/adma.201203348 PMC3832625

[2] Yim, S., Gultepe, E., Gracias, D. H. & Sitti, M. Biopsy using a magnetic capsule endoscope carrying, releasing, and retrieving untethered microgrippers. *IEEE Trans. Biomed. Eng.* **61**, 513–521 (2014). 24108454 10.1109/TBME.2013.2283369 PMC4023810

[3] Choi. A, Gultepe. E & Gracias. D. H. Pneumatic delivery of untethered microgrippers for minimally invasive biopsy, in *13th IEEE International Conference on Control & Automation (ICCA)*, 857–860 (IEEE, 2017). 10.1109/ICCA.2017.8003172 PMC6711395 31456871

[4] Diller, E. & Sitti, M. Three-dimensional programmable assembly by untethered magnetic robotic micro-grippers. *Adv. Funct. Mater.* **24**, 4397–4404 (2014).

[5] Chung, S. E., Dong, X. G. & Sitti, M. Three-dimensional heterogeneous assembly of coded microgels using an untethered mobile microgripper. *Lab Chip* **15**, 1667–1676 (2015). 25714053 10.1039/c5lc00009b

[6] Giltinan, J., Diller, E. & Sitti, M. Programmable assembly of heterogeneous microparts by an untethered mobile capillary microgripper. *Lab Chip* **16**, 4445–4457 (2016). 27766322 10.1039/c6lc00981f

[7] Malachowski, K. et al. Stimuli-responsive theragrippers for chemomechanical controlled release. *Angew. Chem. -Int. Ed.* **53**, 8045–8049 (2014). 10.1002/anie.201311047 PMC4315180 24634136

[8] Zheng, Z. Q. et al. Ionic shape-morphing microrobotic end-effectors for environmentally adaptive targeting, releasing, and sampling. *Nat. Commun.* **12**, 1 (2021). 33462214 10.1038/s41467-020-20697-w PMC7814140

[9] Zhang, J. C. & Diller, E. Tetherless mobile micrograsping using a magnetic elastic composite material. *Smart Mater. Struct.* **25**, 7 (2016).

[10] Ge, Q. et al. Multimaterial 4D printing with tailorable shape memory polymers. *Sci. Rep.* **6**, 11 (2016). 27499417 10.1038/srep31110 PMC4976324

[11] Huang, C. L. et al. A remotely driven and controlled micro-gripper fabricated from light-induced deformation smart material. *Smart Mater. Struct.* **25**, 11 (2016).

[12] Chen, B. K., Zhang, Y. & Sun, Y. Active release of microobjects using a MEMS microgripper to overcome adhesion forces. *J. Microelectromech. Syst.* **18**, 652–659 (2009).

[13] Chen, T., Sun, L. N., Chen, L. G., Rong, W. B. & Li, X. X. A hybrid-type electrostatically driven microgripper with an integrated vacuum tool. *Sens. Actuator A—Phys.* **158**, 320–327 (2010).

[14] Sun, Y. H. et al. Origami-inspired folding assembly of dielectric elastomers for programmable soft robots. *Microsyst. Nanoeng.* **8**, 11 (2022). 35450326 10.1038/s41378-022-00363-5 PMC8971403

[15] Xu, Q. S. Design, fabrication, and testing of an MEMS microgripper with dual-axis force sensor. *IEEE Sens. J.* **15**, 6017–6026 (2015).

[16] Wang, C. et al. Design of a large-range rotary microgripper with freeform geometries using a genetic algorithm. *Microsyst. Nanoengineering* **8**, 14 (2022). 10.1038/s41378-021-00336-0 PMC8733005 35047208

[17] Jia, K. M., Pal, S. & Xie, H. K. An electrothermal tip-tilt-piston micromirror based on folded dual S-shaped bimorphs. *J. Microelectromech. Syst.* **18**, 1004–1015 (2009).

[18] Xiao, L., Ding, Y. T., Wang, P. & Xie, H. K. Analog-controlled light microshutters based on electrothermal actuation for smart windows. *Opt. Express* **28**, 33106–33122 (2020). 33114980 10.1364/OE.405142

[19] Yang, H. et al. A robust lateral shift free (LSF) electrothermal micromirror with flexible multimorph beams. *Microsyst. Nanoeng.* **9** (2023). 10.1038/s41378-023-00570-8 PMC10465609 37654693

[20] Gao, W., Wang, L. L., Wang, X. Z. & Liu, H. Z. Magnetic driving flowerlike soft platform: biomimetic fabrication and external regulation. *ACS Appl. Mater. Interfaces* **8**, 14182–14189 (2016). 27182884 10.1021/acsami.6b03218

[21] Yan, J. et al. Ultracompact single-nanowire-morphed grippers driven by vectorial Lorentz forces for dexterous robotic manipulations. *Nat. Commun.* **14**, 10 (2023). 37355640 10.1038/s41467-023-39524-z PMC10290722

[22] Kim, D. H., Lee, M. G., Kim, B. & Sun, Y. A superelastic alloy microgripper with embedded electromagnetic actuators and piezoelectric force sensors: a numerical and experimental study. *Smart Mater. Struct.* **14**, 1265–1272 (2005).

[23] Li, Y., Liu, Y. J. & Luo, D. Polarization dependent light-driven liquid crystal elastomer actuators based on photothermal effect. *Adv. Opt. Mater.* **9**, 9 (2021).

[24] Han, B. et al. Multi-field-coupling energy conversion for flexible manipulation of graphene-based soft robots. *Nano Energy* **71**, 11 (2020).

[25] Ko, J. et al. Electroosmosis-driven hydrogel actuators using hydrophobic/hydrophilic layer-by-layer assembly-induced crack electrodes. *ACS Nano* **14**, 11906–11918 (2020). 32885947 10.1021/acsnano.0c04899

[26] Li, R. et al. Stimuli-responsive actuator fabricated by dynamic asymmetric femtosecond bessel beam for in situ particle and cell manipulation. *ACS Nano* **14**, 5233–5242 (2020). 32195582 10.1021/acsnano.0c00381

[27] Wani, O. M., Zeng, H. & Priimagi, A. A light-driven artificial flytrap. *Nat. Commun.* **8**, 7 (2017). 28534872 10.1038/ncomms15546 PMC5457518

[28] Zhang, J. C., Onaizah, O., Middleton, K., You, L. D. & Diller, E. Reliable grasping of three-dimensional untethered mobile magnetic microgripper for autonomous pick-and-place. *IEEE Robot. Autom. Lett.* **2**, 835–840 (2017).

[29] Zhang, H. et al. Wireless power transfer to electrothermal liquid crystal elastomer actuators. *ACS Appl. Mater. Interfaces* **15**, 27195–27205 (2023). 37227697 10.1021/acsami.3c03817

[30] Hui, X. S., Luo, J. J., Wang, X. L., Wang, R. & Sun, H. Bimorph electrothermal micro-gripper with large deformation, precise and rapid response, and low operating voltage. *Appl. Phys. Lett.* **121**, 7 (2022).

[31] Zhang, R., Chu, J. K., Wang, H. X. & Chen, Z. P. A multipurpose electrothermal microgripper for biological micro-manipulation. *Microsyst. Technol.* **19**, 89–97 (2013).

[32] Yang. H. Z. et al. An Electrothermal Microcage based on Al-SiO_2_ Bimorph Actuators. *in 37th International Conference on Micro Electro Mechanical Systems (MEMS)*, 725–728 (IEEE, 2024).

[33] Fang. X. et al. Destructive Reliability Analysis of Electromagnetic MEMS Micromirror Under Vibration Environment, IEEE. *J. Sel. Top. Quantum Electron.* **28**, 1–8 (2022).

[34] Tostado. S & Chow. J. Assembly process and solder joint integrity of the metal ball grid array (MBGA/sup TM/) package. *In 46th Electronic Components and Technology Conference*, 1265–1270 (IEEE, 1996).

[35] Ding. M. Z, Wai. L. C, Zhang. S & Rao. V. S. Evaluation of laser solder ball jetting for solder ball attachment process. *In 14th Electronics Packaging Technology Conference (EPTC)*, 23–29 (IEEE, 2012).

