# Supplementary material for: A microgripper based on electrothermal Al–SiO2 bimorphs
Source: Microsyst Nanoeng. 2024 Dec 16;10:195. doi: 10.1038/s41378-024-00821-2 (PMC11649916; doi:10.1038/s41378-024-00821-2)
Supplement: Supplementary file 1 — Supplemental Material File #1 [file 41378_2024_821_MOESM1_ESM.docx]

**Supplementary information**

**A Microgripper Based on Electrothermal Al-SiO_2_ Bimorphs**

**Hengzhang Yang^1,2^, Yao Lu^1,2,3^, Yingtao Ding^1,2^, Ziyue Zhang^1,2^ Anrun Ren^1,2^, Haopu Wang^1,2^, Xiaoyi Wang^1,2,3^, Jiafang Li^4^, Shuailong Zhang^1,2,3*^ and Huikai Xie^1,2,3*^**

^1^School of Integrated Circuits and Electronics, Beijing Institute of Technology, Beijing, CHINA

^2^Engineering Research Center of Integrated Acousto-opto-electronic Microsystems, Ministry of Education of China, Beijing, CHINA

^3^Chongqing Institute of Microelectronics and Microsystems, Beijing Institute of Technology, Chongqing, CHINA

^4^School of Physics, Beijing Institute of Technology, Beijing, CHINA

[shuailong.zhang@bit.edu.cn](mailto:shuailong.zhang@bit.edu.cn)

[hk.xie@bit.edu.cn](mailto:hk.xie@bit.edu.cn)

**Supplementary Materials:**

Figures S1-S12.

Table S1-S4.

Movie S1-S6.

References S1- S9.

**Supplementary Figures**


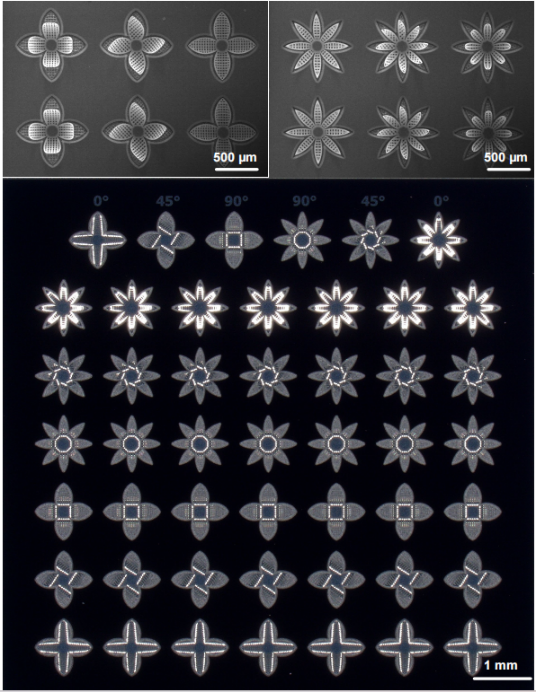


**Figure S1.** Images of an array of actuators with different designs (all on a single silicon wafer).

**
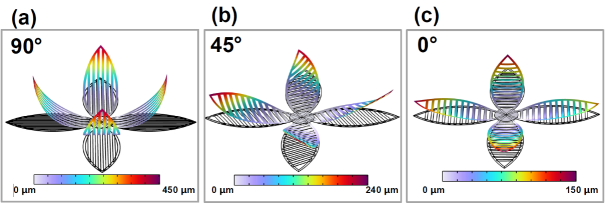
**

**Figure S2. The simulation results of the microgripper.** (a) Simulation result and the 90°-type case. (b) Simulation result and the 45°-type case. (c) Simulation result and the 0°-type case

Three typical bimorph configurations are designed and named 0°-type, 45°-type and 90°-type, respectively. And the curling behaviors of these devices were evaluated using COMSOL finite element simulation software, and the results are shown in Figure S2. To effectively capture the sample, it is necessary to ensure that the actuator curls in the radial direction after the device is released. It is shown that the 90°-type case has the required performance in both bending angle and deforming direction.


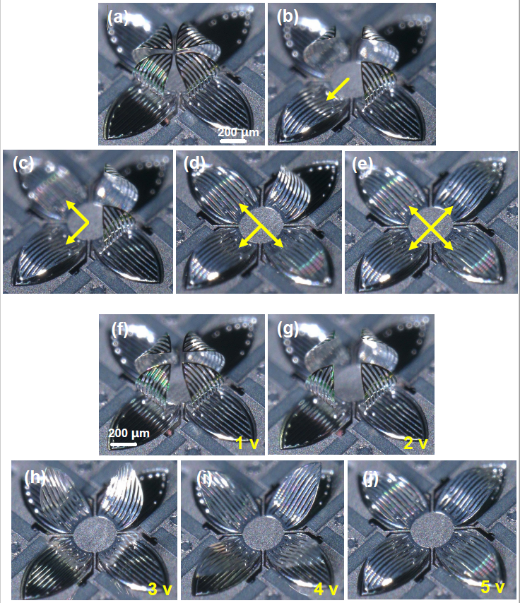


**Figure S3. Optical images of the different working modes of a microgripper.** (a~e). The four actuators are driven in sequence at 5 V. (f~j). The four actuators are driven simultaneously at a driving voltage from 1 V to 5 V.


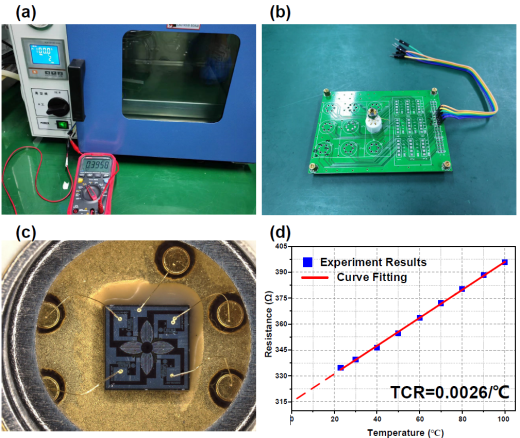


**Figure S4. The experimental method for TCR.** (a) Experimental setup. A temperature-controlled oven is used to set the temperature, and a multimeter is used to measure the resistance at different temperatures. (b) Close view of the PCB board of the microgripper. (c) Close view of the device with Transistor Outline (TO) package. (d) Relationship between the resistance and temperature. The measurement error of the resistance is ±1 Ω. The TCR of the resistance can be calculated as the ratio of the slope to the R-axis intercept of its R-T curve [S1], and the value is calculated to be 0.0026/K.


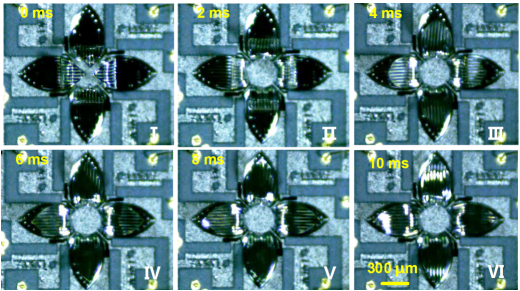


**Figure S5.** Six states of the microgripper during the opening process.


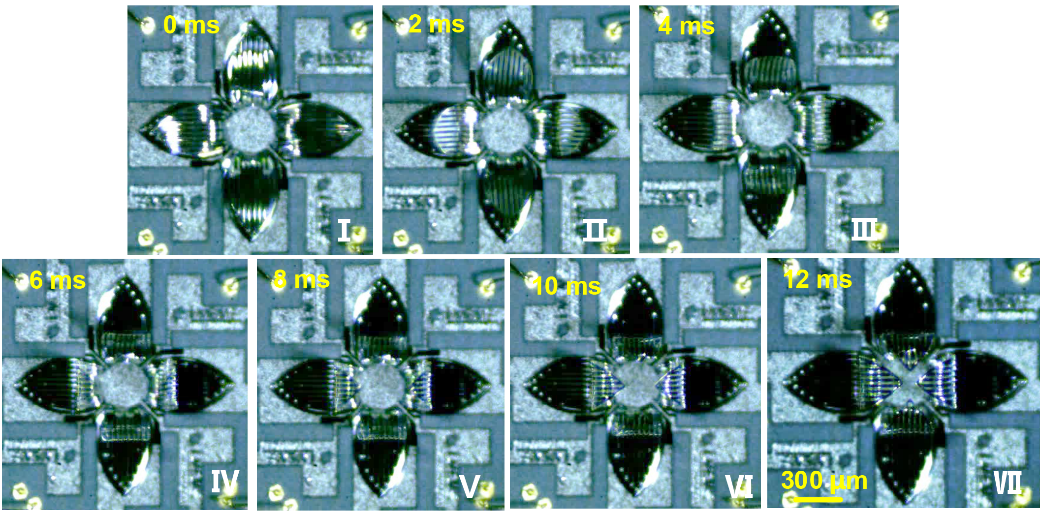


**Figure S6.** Seven states of the microgripper during the closing process.


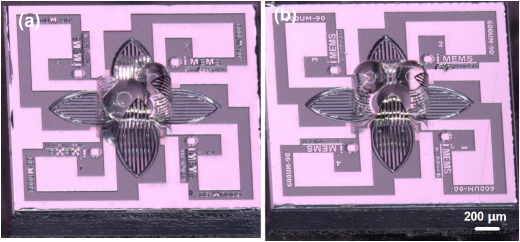


**Figure S7.** **Optical images of multiple PMMA microbeads captured by the microgripper.** (a) Two microbeads with different diameters are captured by the microgripper. (400 μm and 300 μm) (b) Three microbeads with the same diameters are captured by the microgripper (300 μm).


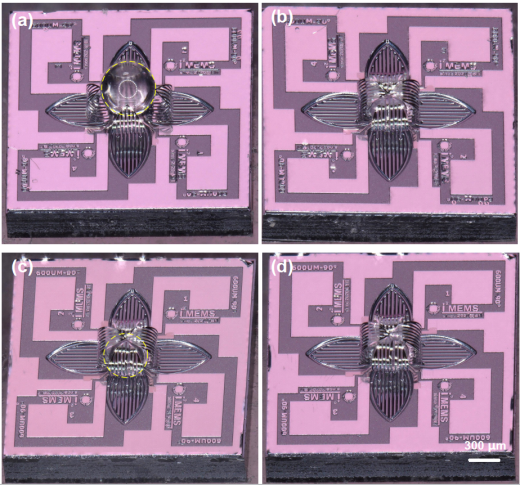


**Figure S8.** **Optical images of the two wrapping modes before and after the vibration tests.** (a~b) Type-A. (c~d) Type-B.

Figure S8(a) shows the shape of the Type-A before the vibration test. Figure S8(b) shows the shape of the Type-A after the vibration test, the microgripper recovers to its original structure under the residual stress. Figures S8(c~d) show the structure of the Type-B before and after the vibration test, respectively. Although the microbead falls from the microgripper, the vibration has little effect on the actuator's mechanical structure and functionality.


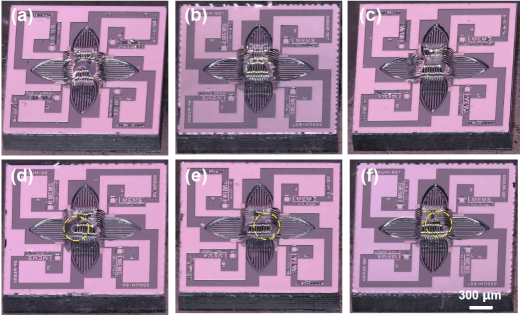


**Figure S9.** **Optical images of the two wrapping modes after impact tests.** (a~c) Type-A. (d~f) Type-B.

Figures S9(a~c) show the structures of the Type-A after impact tests. The actuator is irreversibly deformed under the impact of the microbead. Despite suffering from an acceleration load of 1600g, the microbead is still firmly held by the microgripper (Type-B) with the microbead’s position slightly shifted from its original place, as shown in Figure S9 (d~f).


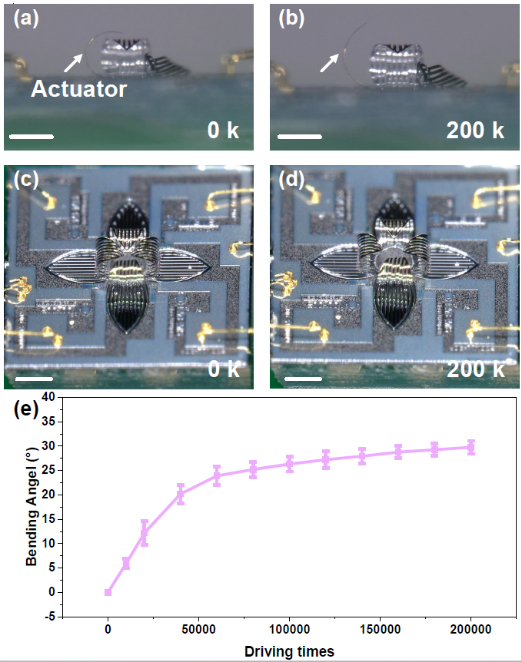


**Figure S10. Longtime driving test of the microgripper.** (a~b) Side-view of one actuator before and after making it open and close 200 thousand times. (c~d) Top-view of the microgripper before and after making it open and close 200 thousand times. (e) The bending angle versus driving times of the actuators. All scale bars in Figure S11 are 400 μm.

To evaluate the stability of the microgrippers, we conducted long-term tests on four devices with a single actuator and four microgrippers. The test was carried out at room temperature and was driven by a sinusoidal signal with a peak-to-peak voltage of 5 V and the frequency was set to 1 Hz. Figure S10(a~b) shows the morphology comparison of a single actuator after 200 thousand consecutive operations. Figure S10(c~d) shows the morphology comparison of the microgripper after 200 thousand consecutive operations. Figure S10(e) shows the relationship between the initial angle of the actuator and driving times. The test results show that after working continuously for 200 thousand times, it is difficult for the microgripper to return to the original closed state, and the actuator angle has changed by about 30° compared to the original state.


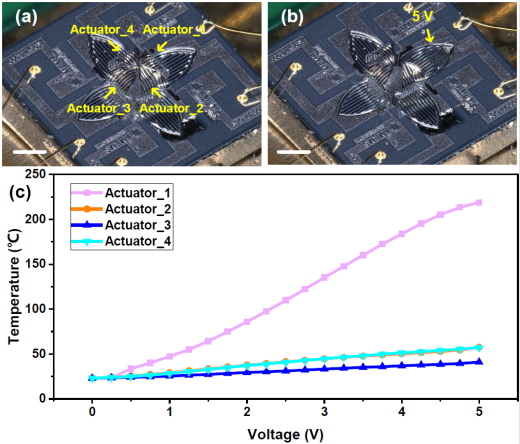


**Figure S11. Thermal crosstalk of the microgripper.** (a) The side view of the microgripper when power-off. (b) The side-view of the microgripper when the Actuator_1 is power-on. (c) The temperature versus applied voltage of four actuators when only Actuator_1 is powered on.

Due to the lack of thermal isolation design, when one of the actuators is heated, the heat will be transferred to other actuators through the substrate, resulting in thermal crosstalk. Figure S11(a-b) shows the morphological changes of the microgripper when a 5 V voltage is applied to Actuator_1 only. Figure S11(c) shows the temperature change of each actuator when only Actuator_1 is heated. The temperatures of the actuators are obtained by measuring the resistance change of the heating resistor in each actuator. With 5 V applied to Actuator_1, the temperature increases of the two actuators adjacent to Actuator_1 reach about 59°C, while the opposing actuator is less affected, with a temperature rise of about 42°C.


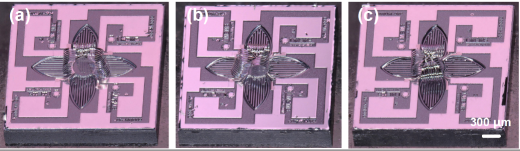


**Figure S12.** Optical images of the damaged microgripper.

**Supplementary Table**

Table S1. Comparison of properties of the used materials

| Material | Young’s modulus (GPa) | CTE (ppm·K^-1^) | Thermal conductivity (W·m^-1^·K^-1^) | Electrical conductivity (S·m^-1^) |
| --- | --- | --- | --- | --- |
| Al [S2] | 70 | 23.1 | 237 | 35.5 × 10^6^ |
| SiO_2_ [S2] | 70 | 0.5 | 1.4 | Insulator |
| Pt [S2] | 168 | 8.8 | 71.6 | 8.9 × 10^6^ |
| Polyimide [S3] | 2.3 | 36 | 0.14 | Insulator |

Table. S2 Key parameters of the microgripper

| **Structural parameter** | **Value** |
| --- | --- |
| Device footprint | 2.4 mm ×2.4 mm |
| Diameter of the center plate (D) | 400 μm |
| Overall length of the actuator (L) | 600 μm |
| Width of individual bimorph beams (W_b_) | 20 μm |
| Width of the gap between two adjacent bimorphs (W_g_) | 20 μm |
| Thickness of the structural Al layer | 500 nm |
| Thickness of the structural SiO_2_ layer | 400 nm |
| Thickness of the insulation SiO_2_ layer | 100 nm |
| Thickness of the Pt layer | 100 nm |

Table S3. The resistance of the ten measured actuators

| Number | 1 | 2 | 3 | 4 | 5 | 6 | 7 | 8 | 9 | 10 |
| --- | --- | --- | --- | --- | --- | --- | --- | --- | --- | --- |
| Resistance  (Ω) | 332 | 332 | 319 | 332 | 325 | 336 | 335 | 371 | 367 | 378 |

Table S4. Performance comparison of the microgripper reported in this paper and other works

| Work | Material | Bending  Angle (°) | Working  Temperature （℃） | Response  Time (ms) | Power  Consumption (mW) | Actuator  Length  (μm) |
| --- | --- | --- | --- | --- | --- | --- |
| [S3] | DLC/Ni | 90 | 430 | \ | 20 | 160 |
| [S4] | DLC/TiNi | 60 | 80 | \ | \ | 150 |
| [S5] | Cr/VO_2_ | 43 | 80 | 0.17 | 1.6 | 118 |
| [S6] | Polymers | 360 | 5/45 | 55×10^3^ | \ | ＞10^4^ |
| [S7] | Liquid crystal elastomer | ＞200 | 70 | 2×10^3^ | 1×10^3^ | ＞10^4^ |
| [S8] | Al/NiTi | ＜50 | 90 | 10 | 76 | 100 |
| [S9] | Liquid crystal elastomer | 360 | 130 | 12×10^3^ | \ | ＞10^4^ |
| This work | Al/SiO_2_ | ＞100 | 200 | 10 | 48 | 600 |

**Supplementary Movies**

**Movie S1**: The detailed deformation process of the microgripper. The shooting speed of the high-speed camera is 2000 frames per second, and the play speed of the video is 1/30 of the actual speed.

**Movie S2**: The detailed failure process of Type A during a vibration test. The acceleration of the plate is 5g.

**Movie S3**: The detailed manipulation process of a solder bead with a diameter of 400 μm.

**Movie S4**: The detailed manipulation process of a solder bead with a diameter of 200 μm.

**Movie S5**: The long-time driving test of a representative microgripper (top view).

**Movie S6**: The long-time driving test of a representative actuator (side view).

**Reference**

S1 Xiao, L., Ding, Y. T., Wang, P. & Xie, H. K. Analog-controlled light microshutters based on electrothermal actuation for smart windows. *Opt. Express* **28**, 33106-33122 (2020).

S2 Pal, S. & Xie, H. K. Fabrication of robust electrothermal MEMS devices using aluminum-tungsten bimorphs and polyimide thermal isolation. *J. Micromech. Microeng.* **22**, 14 (2012).

S3 Luo, J. K. *et al.* Fabrication and characterization of diamond-like carbon/Ni bimorph normally closed microcages. *J. Micromech. Microeng.* **15**, 1406-1413 (2005).

S4 Fu, Y. Q. *et al.* A shape memory microcage of TiNi/DLC films for biological applications. *J. Micromech. Microeng.* **18**, 8 (2008).

S5 Liu, K. *et al.* Giant-Amplitude, High-Work Density Microactuators with Phase Transition Activated Nanolayer Bimorphs. *Nano Lett.* **12**, 6302-6308 (2012).

S6 Li, J. *et al.* Highly Bidirectional Bendable Actuator Engineered by LCST-UCST Bilayer Hydrogel with Enhanced Interface. *ACS Appl. Mater. Interfaces* **12**, 55290-55298 (2020).

S7 Ma, B., Xu, C. T., Cui, L. S., Zhao, C. & Liu, H. Magnetic Printing of Liquid Metal for Perceptive Soft Actuators with Embodied Intelligence. *ACS Appl. Mater. Interfaces* **13**, 5574-5582 (2021).

S8 Hui, X. S., Luo, J. J., Wang, X. L., Wang, R. & Sun, H. Bimorph electrothermal micro-gripper with large deformation, precise and rapid response, and low operating voltage. *Appl. Phys. Lett.* **121**, 7 (2022).

S9 Zhang, H. *et al.* Wireless Power Transfer to Electrothermal Liquid Crystal Elastomer Actuators. *ACS Appl. Mater. Interfaces* **15**, 27195-27205 (2023).
